# Amino Acid Prodrugs: An Approach to Improve the Absorption of HIV-1 Protease Inhibitor, Lopinavir

**DOI:** 10.3390/ph7040433

**Published:** 2014-04-10

**Authors:** Mitesh Patel, Nanda Mandava, Mitan Gokulgandhi, Dhananjay Pal, Ashim K. Mitra

**Affiliations:** Division of Pharmaceutical Sciences, School of Pharmacy, University of Missouri-Kansas City, 2464 Charlotte Street, Kansas City, MO 64108, USA

**Keywords:** lopinavir, LPV, prodrug, uptake, transport, permeability, efflux, P-gp, MRP2

## Abstract

Poor systemic concentrations of lopinavir (LPV) following oral administration occur due to high cellular efflux by P-glycoprotein (P-gp) and multidrug resistance-associated proteins (MRPs) and extensive metabolism by CYP3A4 enzymes. In this study, amino acid prodrugs of LPV were designed and investigated for their potential to circumvent efflux processes and first pass effects. Three amino acid prodrugs were synthesized by conjugating isoleucine, tryptophan and methionine to LPV. Prodrug formation was confirmed by the LCMS/MS and NMR technique. Interaction of LPV prodrugs with efflux proteins were carried out in P-gp (MDCK-MDR1) and MRP2 (MDCK-MRP2) transfected cells. Aqueous solubility studies demonstrated that prodrugs generate higher solubility relative to LPV. Prodrugs displayed higher stability under acidic conditions and degraded significantly with rise in pH. Uptake and transport data suggested that prodrugs carry significantly lower affinity towards P-gp and MRP2 relative to LPV. Moreover, prodrugs exhibited higher liver microsomal stability relative to LPV. Hence, amino acid prodrug modification might be a viable approach for enhancing LPV absorption across intestinal epithelial and brain endothelial cells which expresses high levels of P-gp and MRP2.

## 1. Introduction

LPV is a protease inhibitor (PI) that is commonly indicated in highly active antiretroviral therapy (HAART) for the treatment of HIV infection. Inclusion of PIs in HAART has significantly improved clinical outcomes in HIV infected patients. However, a major problem associated with PIs is their inability to attain higher systemic concentrations following oral administration. LPV alone displayed poor oral bioavailability primarily due to high P-gp and MRP2 mediated efflux [1] and hepatic metabolism [2]. P-gp and MRP2 are predominantly expressed on the villus tip of enterocytes which is the primary absorption site for orally administered therapeutic agents. Active secretion of LPV back into the intestinal lumen significantly diminishes its transport thereby resulting in lower bioavailability. Moreover, LPV has been reported to be extensively metabolized by CYP3A4 enzymes in the liver [2,3]. The coordinated function of efflux processes and CYP3A4 enzymes results in low to variable oral absorption of LPV. Importantly, high expression of P-gp and MRP2 at the blood-brain barrier (BBB) may be a major factor limiting brain transport of LPV from systemic circulation. As a result of negligible brain LPV absorption, the virus harboring in brain parenchyma remains unchecked resulting in a HIV-1 sanctuary site and AIDS-related dementia. Hence to achieve therapeutic concentrations, large doses of PIs are administered which results in unacceptable systemic side effects [4].

Various strategies have been employed to improve oral and brain absorption of P-gp and MRP2 substrates. One of the most common approaches employed have been the co-administration of P-gp and MRP2 substrates with efflux inhibitors. However, systemic administration of high doses of inhibitors required to inhibit P-gp and/or MRP2 may result in unacceptable toxicities. Moreover, these efflux proteins are also expressed in normal tissues and extrude xenobiotics and cytotoxic molecules thereby providing cellular protection. Therefore, inhibition of these efflux processes might result in cellular side effects. Prodrug based delivery strategies capable of circumventing P-gp and MRP2 mediated efflux could be valuable in such circumstances. Transporter targeted prodrug modification is one such promising strategy where the ligand coupled drugs can bypass efflux pumps by binding to influx transporters highly expressed on the intestinal epithelium and/or BBB.

Peptide transporters have been widely targeted with prodrugs to improve absorption of poorly permeable but highly potent drugs. These transporters have become an attractive target as they can transport compounds with diverse chemical modification in their substrate structure. Moreover, these carrier transporters possess high substrate capacity and broad specificity. Recently, L-valyl and L-isoleucyl amino acid prodrugs of guanidine oseltamivir carboxylate have been examined for their affinity towards peptide transporters using Gly-Sar as a model PepT1 substrate [5]. These prodrugs significantly diminished Gly-Sar uptake and displayed more than 30-fold higher affinity towards PepT1 transporter relative to parent drug, guanidine oseltamivir carboxylate. Similarly, 5'-L-isoleucyl and 5'-L-valyl monoester prodrugs of floxuridine generated 8 to 11-fold higher Caco2 permeability relative to floxuridine [6]. Importantly, these prodrugs exhibited 8 to 19-fold higher uptake in HeLa/PepT1 cells relative to floxuridine. Moreover, valine and isoleucine prodrugs have displayed high efficacy in improving intestinal transport of zanamivir [7]. Valine and isoleucine prodrugs have produced approximately 9 and 2-fold higher Caco2 permeability relative to zanamivir. In addition, peptide transporter has been previously targeted in our laboratory with amino acid and/or peptide prodrugs to improve ocular absorption of acyclovir [8] and ganciclovir [9,10,11,12] and intestinal transport of LPV [13] and saquinavir [14]. Interestingly, amino acid prodrugs of quinidine, *i.e.*, valine-quinidine and isoleucine-quinidine have demonstrated high potential in circumventing P-gp mediated cellular efflux [15,16]. Based on these observations, we anticipate that peptide transporter might serve as a valuable target for improving the absorption of LPV prodrugs.

In this study, isoleucine-LPV (Ile-LPV), tryptophan-LPV (Trp-LPV) and methionine-LPV (Met-LPV) prodrugs were synthesized with a simple esterification process. These prodrugs were designed to examine the effect of amino acids with different side chains (aliphatic or aromatic) in circumventing P-gp and MRP2-mediated cellular efflux of LPV. Moreover, Ile-LPV was specifically designed as it has been reported that zidovudine prodrugs containing hydrophobic beta-branched amino acids exhibit higher chemical and plasma stability [17]. Prodrugs were synthesized and identified using LCMS/MS analysis. Intracellular accumulation and transport studies in MDCK-MDR1 and MDCK-MRP2 cell lines were conducted to investigate the affinity of prodrugs towards efflux proteins such as P-gp and MRP2, which are highly expressed at intestinal epithelium and BBB. The selected cell lines have been previously employed as an *in vitro* cell culture models for studying interaction with efflux proteins [1,18]. Cyclosporine A (P-gp substrate/inhibitor), GF 120918 (P-gp inhibitor) and MK 571 (MRP2 inhibitor) were also employed to delineate uptake and transport mechanisms. Rat liver microsomes were employed to determine the extent of LPV and prodrug metabolism.

## 2. Experimental Section

### 2.1. Materials

Unlabeled LPV was a generous gift from Abbott Laboratories Inc. (North Chicago, IL, USA) [3H]-LPV (1 Ci/mmol) was purchased from Moravek Biochemicals (Brea, CA, USA) and used at 0.25 µCi/mL. MDCK cells, retrovirally transfected with human *MDR1* cDNA (MDCK-MDR1) and human MRP2 (MDCKII-MRP2) and wild type MDCKII (MDCK WT) cells were generously provided by Drs. A. Schinkel and P. Borst (Netherlands Cancer Institute, Amsterdam, The Netherlands). The growth medium Dulbecco’s modified Eagle’s Medium (DMEM), trypsin/EDTA and non-essential amino acids were obtained from Gibco (Invitrogen, Grand Island, NY, USA). Fetal bovine serum (FBS) was obtained from Atlanta biological. Sprague Dawley rat liver microsomes were purchased from XenoTech LLC (Lenexa, KS, USA). Penicillin, triton X-100, HEPES, D-glucose, streptomycin, sodium bicarbonate, cyclosporin A, GF 120918, MK 571, Boc-methionine, Boc-tryptophan, Boc-isoleucine, dichloromethane, ethyl acetate, 4-(*N*,*N*-dimethylamino)pyridine (DMAP), glucose, dicyclohexylcarbodiimide (DCC) sodium chloride (NaCl), potassium chloride (KCl), sodium phosphate (Na_2_ HPO_4_), potassium phosphate (KH_2_PO_4_), calcium chloride (CaCl_2_), magnesium sulfate (MgSO_4_) and other chemicals were purchased from Sigma Chemical Co. (St. Louis, MO, USA). DMSO and methanol (HPLC grade) were purchased from Fisher Scientific Co. (Pittsburgh, PA, USA). Premium siliconized microcentrifuge tubes were purchased from MIDSCI (St. Louis, MO, USA) Culture flasks (75 and 25 cm^2^) and 12-well plates (3.8 cm^2^ growth area/well) were obtained from Costar (Bedford, MA, USA). All chemical agents procured were of special reagent grade and utilized without any further purification.

### 2.2. Methods

#### 2.2.1. Cell Culture

MDCK-WT, MDCK-MRP2 and MDCK-MDR1 cells (passage 8–10) were grown in 75 cm^2^ tissue culture flasks in DMEM medium supplied with high glutamine and glucose concentrations. Culture medium was supplemented with 10% FBS (heat inactivated), penicillin (100 units/mL) and streptomycin (100 µg/mL) and maintained at pH 7.4. Cells were incubated at 37 °C in an atmosphere of 5% CO_2_ and 90% relative humidity. The medium was replaced every alternate day until cells reached 70%–80% confluency (5–7 days).

#### 2.2.2. Solubility Study in Distilled Deionized Water (DDW)

Aqueous solubility study of prodrugs was performed according to the published protocol from our laboratory [13]. Saturated solution of each prodrug was prepared in DDW in siliconized tubes. Tubes were placed in shaking water bath at room temperature (RT) for 24 h. Tubes were centrifuged at 10,000 rpm for 10 min to separate undissolved prodrugs. The supernatant was separated, filtered through 0.45 µm membrane (Nalgene syringe filter) and analyzed with HPLC after appropriate dilutions.

#### 2.2.3. Buffer Stability Studies

Chemical hydrolysis study of prodrugs was carried out according to the previously published protocols from our laboratory [19]. Degradation rate constant and half-life (t_1/2_) of prodrugs were determined at various pH. Approximately, 50 µM prodrug solution was added in 1.5 mL of DPBS adjusted to pH 5, 6 and 7.4. Microcentrifuge tubes were placed in shaking water bath (60 rpm) at 37 °C. Samples (100 µL) were withdrawn at predetermined time points and stored at −80° C until further analysis. Prodrug concentrations remaining were plotted *versus* time in order to calculate degradation rate constants.

#### 2.2.4. Cytotoxicity Studies

Cytotoxicity of prodrugs was determined in MDCK-WT cells with MTS assay based cytotoxicity kit. Briefly, cells were grown overnight in 96 well plates at a density of 20,000 cells per well prior to drug and prodrug addition. Medium was aspirated and replaced with 100 μL of serum free solution containing serial dilutions of test compounds (5–250 μM). Cells were then incubated for 4 h at 37 °C_._ Following incubation, 20 μL of MTS stock solution was added to each well and incubated for 2 h at 37 °C. Cell viability was determined by measuring absorbance at 485 nm with the help of a plate reader (BioRad, Hercules, CA, USA). The quantity of formazan product as measured by absorbance is directly proportional to the number of viable cells in test samples.

#### 2.2.5. Uptake Studies

Confluent cells were then trypsinized and plated at a density of 3 × 10^6^ cells in 12 well culture plates. Medium was replaced every alternate day until cells reached confluency (6–7 days). Cellular uptake studies were performed on confluent cell monolayers according to previously published protocol from our laboratory [20,21]. Briefly, medium was removed and cell monolayer was washed with 2 mL of DPBS (130 mM NaCl, 0.03 mM KCl, 7.5 mM Na_2_HPO_4_, 1.5 mM KH_2_PO_4_, 1 mM CaCl_2_, 0.5 mM MgSO_4_, and 5 mM glucose, pH 7.4) three times at 37 °C (each wash of 10 min). Uptake studies were initiated by incubating cells with [3H]-LPV in DPBS at 37 °C for 30 min. Following incubation, radioactive solution was quickly removed and plates were washed with ice-cold stop solution (210 mM KCl, 2 mM HEPES, pH of 7.4) to arrest the uptake process. One mL of lysis buffer (0.1% Triton-X solution in 0.3% NaOH) was added to each well and plates were stored overnight at RT. Later, 500 µL solutions were transferred to scintillation vials containing 3 mL of scintillation cocktail. The radioactivity associated with cells was analyzed with a scintillation counter (Beckman Instruments Inc., Model LS-6500; Fullerton, CA, USA). Uptake rate was normalized to protein count which was quantified with a BioRad protein estimation kit. To study the effect of efflux inhibitors, cells were treated with GF 120918 (MDCK-MDR1) and MK 571 (MDCK-MRP2) prior to the initiation of the uptake experiment.

#### 2.2.6. Transport Studies

Transepithelial transport studies were carried out according to the protocols published from our laboratory [19]. Transwell^®^ inserts (0.4 µm pore size, 12 mm insert) with transparent polyester membranes were employed to study transepithelial transport of LPV and prodrugs. Transwell^®^ inserts were coated with type 1 rat tail collagen (100 µg/cm^2^). Cells were seeded at a density of 250,000 cells per well. Transport studies were conducted across MDCK-MDR1 and MDCK-MRP2 cells in presence and absence of efflux inhibitors for a period of 3 h at 37 °C. Prior to the initiation of transport study, cell monolayer integrity was evaluated by monitoring transepithelial electric resistance (TEER), with an EVOM (epithelial volt ohmmeter from World Precision Instruments, Sarasota, FL, USA). TEER values of the cell monolayer were approximately 250 Ω*cm^2^. Monolayers were washed thoroughly with DBPS (pH7.4) for 15 min at 37 °C, three times. For determining A–B permeability, 0.5 mL LPV or prodrug solution (25 µM) was added to the apical chamber of 12-well Transwell^®^ plates. Similarly, 1.5 mL LPV or prodrug solution (25 µM) was added to the basolateral chamber to determine B-A permeability. At predetermined time points (30, 60, 120 and 180 min), 100 µL sample was withdrawn from the receiving chamber and replaced with fresh DPBS to maintain sink conditions. Samples were stored at −80 °C until further analysis. To determine the effect of efflux inhibitors, cells were treated GF 120918 (MDCK-MDR1) and MK 571 (MDCK-MRP2) prior to initiation of the transport study.

#### 2.2.7. Rat Microsomal Stability Studies

Rat liver microsomes were employed to determine the substrate affinity of LPV and prodrugs towards CYP3A4. One mL of microsomal solution was generated by adding microsomal protein (0.3 mg/mL), magnesium chloride (5 mM), glucose 6-phosphate (5 mM), b-NADP1 (1 mM), and glucose 6-phosphate dehydrogenase (1 U/mL) in phosphate buffer (100 mM, pH 7.4). LPV or prodrugs was added to the microsomal solution and incubated at 37 °C for 5 min. The metabolic reaction was initiated by adding the NADPH generating system. NADPH generating system was freshly prepared by adding G-6-P, G-6-P DH and NADP in DDW. At predetermined time points, metabolic reaction was arrested by adding equal volumes of ice-cold acetonitrile to the sample. Samples were stored at −80 °C until further analysis by LCMS/MS technique. Both time and concentration dependent stability studies of LPV and prodrugs were conducted. 

##### 2.2.7.1. Time Dependent Stability Studies

For time dependent metabolism studies, LPV or prodrugs (25 μM) were incubated with activated liver microsomal solution. This study was performed according to the previously published protocols from our laboratory [19]. At predetermined time points, about 100 μL of samples were withdrawn and equal volume of acetonitrile was added to stop the degradation process. Degradation study was conducted for a period of 2 h. Samples were stored at −80 °C until further analysis.

##### 2.2.7.2. Concentration Dependent Degradation Studies

For concentration dependent degradation studies, various concentrations of LPV or prodrugs (1–20 μM) were incubated with activated microsomal solution for 5 min. Enzymatic degradation was arrested by adding equal amount of acetonitrile and samples were stored in −80 °C until further analysis.

### 2.3. Data Analysis

#### 2.3.1. HPLC Analysis

Aqueous solubility and buffer stability samples were measured with reversed phase HPLC technique. Waters 515 pump (Waters, Milford, MA, USA) connected to a UV detector (Absorbance Detector Model UV-C, RAININ, Dynamax, Palo Alto, CA, USA) was employed. A C(18) Kinetex column (100 × 4.6 mm, 2.6 µ; Phenomenex, Torrance, CA, USA) was pumped with mobile phase (1:1) acetonitrile and water (0.1% trifluoroacetic acid) at a flow rate of 0.3 mL/min. Analytes were detected with UV detection at a wavelength of 210 nm. Ile-LPV, Met-LPV and Trp-LPV eluted at approximately 9.2, 8.0 and 10.2 min, respectively.

#### 2.3.2. Sample Preparation for LCMS/MS Analysis

Transport samples of LPV and prodrugs were analyzed with a fast and sensitive LC–MS/MS method. Samples were extracted with liquid–liquid extraction technique. About, 50 μL of verapamil (200 ng) was added as an internal standard to each sample. Analytes were extracted by vortexing with 800 μL water saturated ethyl acetate for 2.5 min. Following centrifugation at 10,000 rpm for 7 min, approximately 600 µL of ethyl acetate layer was collected and evaporated under reduce pressure for 45 min. The residue was reconstituted in 100 μL of acetonitrile (80%) and water (20%) containing 0.1% formic acid. Ten microliters of reconstituted samples were injected in LCMS/MS. Similarly, standard solutions were extracted from DPBS and quantified with LCMS/MS.

#### 2.3.3. LCMS/MS Analysis

To analyze transport samples, QTrap^®^ LCMS/MS mass spectrometer (Applied Biosystems, Foster City, CA, USA) connected to Agilent 1100 Series quaternary pump (Agilent G1311A), vacuum degasser (Agilent G1379A) and autosampler (Agilent G1367A, Agilent Technology Inc., Palo Alto, CA, USA) was employed. Chromatographic separation of analytes was obtained with XTerra^®^MS C18 column 50 mm × 4.6 mm, 5.0 μm (Waters, Milford, MA, USA). Mobile phase (80% acetonitrile and 20% water containing 0.1% formic acid) was pumped at 0.3 mL/min flow rate and chromatographs are obtained over 4 min. LPV and verapamil eluted at 3 and 1.7 min, respectively. Ile-LPV, Met-LPV and Trp-LPV eluted within 1.7–1.8 min, respectively.

Electrospray ionization in the positive mode was employed and analytes of interest were detected using multiple-reaction monitoring (MRM) mode. Precursor ion of analytes was determined from spectra which are generated with the infusion of standard solutions to the electrospray source with the help of an infusion pump. Each of these precursor ions was then subjected to collision-induced dissociation to produce product ions. Precursor/product ions observed for LPV and verapamil were 629.398/155.183 and 455.020/150.050, respectively. Precursor/product ions observed for Ile-LPV, Met-LPV and Trp-LPV were 742.489/155.192, 760.547/155.159 and 815.499/155.151, respectively.

We also optimized the turbo ion spray setting and collision gas pressure (ion spray voltage: 2500 V, temperature: 350 °C, collision gas: 8 psi, curtain gas: 30 psi). MS/MS was performed using nitrogen as collision gas. Other operational parameters optimized included declustering potential (DP): 80 V; collision energy (CE): 62 V; entrance potential (EP) 8.5 V; and collision cell exit potential (CXP) 4 V. These parameters were generated by shooting analytes in quantitative optimization mode. Each analyte of interest produced with MRM method showed a significant linearity up to ng range. With this method, rapid and reproducible results were successfully obtained.

#### 2.3.4. Permeability Analysis

Transepithelial permeability was determined by plotting cumulative amount of analyte transported against time. Linear regression of analyte transported as a function of time generated rate of transport (dM/dt) which was divided by the cross-sectional area (A) available for transport to generate steady-state flux as denoted in Equation (1):

Flux = (dM/dt)/A
(1)

Transepithelial permeability was calculated by normalizing the steady-state flux with the donor concentration (Cd) according to Equation (2):

Transepithelial permeability = flux/Cd
(2)

#### 2.3.5. Statistical Analysis

Uptake, transport and buffer stability results were expressed as mean ± S.D. Student’s *t*-test was applied to determine the statistical significance among groups. A difference between the mean values was considered to differ significantly if the *p* value is ≤0.05.

### 2.4. Synthesis and Identification of LPV Prodrugs

#### 2.4.1. Synthesis of LPV Prodrugs

LPV prodrugs were synthesized according to previously published protocols with minor modifications [22]. Briefly, commercially available Boc-isoleucine (0.37g, 1.6 mmol) and DCC (0.5 g, 2.4 mmol) were dissolved in 10 mL anhydrous dichloromethane. The mixture (mixture 1) was stirred for 1 h at 0 °C under nitrogen atmosphere. In a separate round bottom flask, LPV (0.5 g, 0.8 mmol) and DMAP (0.2 g, 1.6 mmol) were dissolved in 5 mL anhydrous dichloromethane and stirred for 15 min at RT. This mixture is then added drop wise to mixture 1 and stirred for 48 h at RT. The reaction mixture was analyzed by TLC using ethyl acetate and liquid chromatography-mass spectrometry (LCMS) to ensure complete conversion of reactants to product. The mixture was filtered and solvent was removed under reduced pressure at RT to obtain crude product. The product Boc-isoleucine-LPV was purified by silica gel column chromatography using ethyl acetate. The eluent was evaporated under reduced pressure which yielded an oily crude product. It was then kept under reduced pressure overnight to obtain dry product. The yield was 80%.

#### 2.4.2. Deprotection of the N-Boc Group

**Scheme 1:**
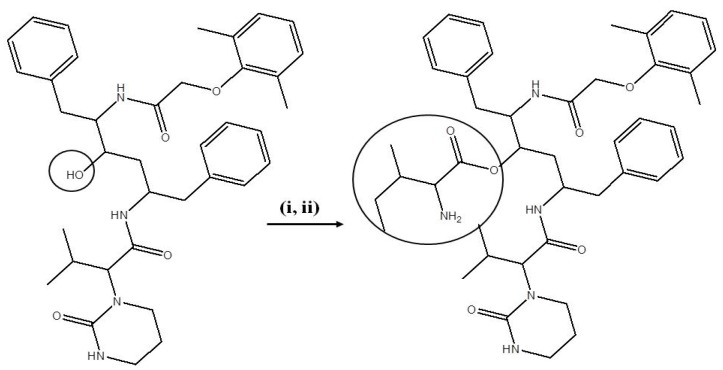
Synthesis scheme of Ile-LPV prodrug.

Boc-isoleucine-LPV was treated with approximately 60% trifluoroacetic acid (TFA) in dichloromethane at 0 °C for about 1 h. The solvent was quickly evaporated under reduced pressure to obtain oily crude product. The product Isoleucine-LPV (Ile-LPV) was purified by recrystallization in cold diethyl ether. This oily product was dissolved in methanol and evaporated under reduced pressure at RT to obtain a final dried powder with a 90% yield. The final product (prodrug) was then stored in −20 °C until further use. The reaction scheme for the synthesis of Ile-LPV is shown in Scheme 1. Met-LPV and Trp-LPV prodrugs were synthesized using similar procedure and reaction conditions.

*Reagents and conditions*: (i) Boc-isoleucine, DCC in DCM: 1 h at 0 °C; LPV, DMAP in DCM: 15 min at RT and mixture stirred for 48 h at RT. (ii) 60% TFA in DCM: 1 h at 0 °C.

### 2.5. Identification and Purity of Prodrugs

Prodrugs were characterized with LCMS/MS, TLC and NMR analysis. A hybrid triple quadrupole-linear ion trap mass spectrometer (QTrap^®^ LC/MS/MS spectrometer—Applied Biosystems) was employed under enhanced mass mode to identify prodrugs. LC/MS (*m/z*); [M+H]^+^ observed for Ile-LPV, Met-LPV and Trp-LPV were 742.5, 760.5 and 815.5, respectively. The purity of prodrugs was determined using TLC and HPLC analysis. For TLC analysis, ethyl acetate was used as the solvent system. For HPLC analysis, C(18) Kinetex column (100 x 4.6 mm, 2.6 µ; Phenomenex, Torrance, CA, USA) was employed. Two different solvents were used: A) acetonitrile and B) water. Both solvents contained 0.1% formic acid. The gradient employed was 0% A to 100% A for 30 min, followed by 100% A for another 10 min. Total runtime was 40 min. The flow rate of solvent system was 0.4 mL/min. The wavelength used for prodrug detection was 210 nm. Purity of all prodrugs was observed to be greater than 95%. ^1^H-NMR ((CD_3_)_2_S=O) analysis was performed to detect the main characteristic peaks supporting the formation of prodrugs. The ppm values (*δ*) observed are (a) Ile-LPV: 0.78 [(CH_3_)_2_-CH, 6H], 0.82 (CH_3_-CH_2_, 3H), 0.90 (CH_3_-CH, 3H), 1.00 (CH_2_-CH, 2H), 6.35 (NH-CO-N, 1H), 6.90–7.10 (benzene, 3H), 7.16–7.3 (benzene, 10H), 8.45 (NH-CO, 1H); (b) Met-LPV: 0.70 [(CH_3_)_2_-CH, 6H], 0.82 (CH_3_-S), 1.22 (CH_2_-CH_2_, 2H), 1.45 (CH_2_-CH-NH_2_, 2H), 3.60 (NH_2_-CH, 2H), 6.35 (NH-CO-N, 1H), 6.90-7.10 (benzene, 3H), 7.16–7.30 (benzene, 10H), 8.60 (NH-CO, 1H); (c) Trp-LPV: 0.7 [(CH_3_)_2_-CH, 6H], 0.82 (CH_2_-CH-NH_2_, 2H), 3.65 (NH_2_-CH, 2H), 6.35 (NH-CO-N, 1H), 6.90–7.10 (benzene, 3H), 7.16–7.30 (benzene, 14H), 8.45 (NH-CO, 1H), 11.1 (NH-indole, 1H).

## 3. Results and Discussion

### 3.1. Solubility in DDW

Solubility values of prodrugs in DDW for Ile-LPV, Met-LPV and Trp-LPV were 625 ± 27, 362 ± 21 and 274 ± 11 µg/mL, respectively. These values were about 15, 9 and 7- fold higher relative to LPV (40 ± 8 µg/mL). Currently, LPV is administered to HIV-1 infected patients in the form of approximately 40% v/v alcoholic solution as it is practically insoluble in water. Such high alcohol concentrations may not be suitable for pediatric patients. Moreover, ethanol has been reported to augment CYP3A activity [23]. An enhancement in CYP3A levels might play a significant contributory role in LPV metabolism. In contrast, prodrugs might offer potential advantages due to their higher solubility relative to LPV.

### 3.2. Buffer Stability Studies

Stability of prodrugs was determined in DPBS at various pH values *i.e.*, 4, 5.5 and 7.4. Results obtained from this study are summarized in Table 1. Prodrugs degraded rapidly at higher pH relative to lower pH. Degradation rate constant at pH 4 was about 20 to 25- fold lower relative to pH 7.4. This result suggested that alkaline hydrolysis of prodrugs might be significantly rapid relative to acidic hydrolysis. Degradation half lives of Ile-LPV, Met-LPV and Trp-LPV at pH 7.4 ranged between 5.0−6.5 h. Such lower half lives at pH 7.4 suggest that a small amount of LPV may be regenerated from prodrugs during the uptake and transport process. For uptake and transport experiments, we selected physiological pH, *i.e.*, 7.4.

**Table 1:** Degradation half lives (h) of prodrugs at various pH values.

Prodrugs	pH 4	pH 5.5	pH 7.4
Ile-LPV	101 ± 29	53 ± 10	6.4 ± 0.3
Met-LPV	119 ± 6	55 ± 6	6.2 ± 0.4
Trp-LPV	123 ± 14	50 ± 9	5.4 ± 0.3

### 3.3. Cytotoxicity Studies

**Figure 1:**
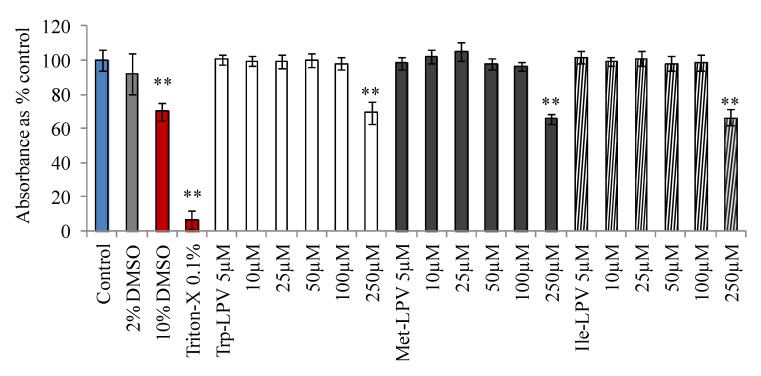
Cytotoxicity result obtained in MDCKII-WT cells following 4 h incubation with prodrugs, Trp-LPV (empty bars), Met-LPV (filled bars) and Ile-LPV (striped bar). Each data point is expressed as mean ± standard deviation (n = 8). Absorbance is expressed as percentage of control (serum/drug free medium). Asterisk (**) represents significant difference from the control (*p* < 0.01).

Cytotoxicity of LPV and prodrugs was determined in MDCK-WT cells with MTT assay. Medium having no drug or organic solvents was used as control. DMSO (10%) or Triton-X (0.1%) containing medium was selected as positive control. Results obtained from this study are demonstrated in Figure 1. Medium containing 2% DMSO did not generate any cytotoxic effects at the end of 4 h incubation. Hence, prodrug concentrations were prepared in such a way that percentage of DMSO in the final solution was less than 2%. However, medium containing 10% DMSO (30% reduction in absorbance) and 0.1% Triton-X (90% reduction in absorbance) produced cytotoxic effects to MDCK-WT cells. Prodrugs did not display any cell cytotoxicity in the concentration range 5–100 µM. However, LPV at 100 µM generated cytotoxic effects. About 23% reduction in the absorbance relative to control was observed when cells were incubated with 100 µM LPV (data not shown). Interestingly at 100 µM, none of the prodrugs displayed any cytotoxicity to MDCK-WT cells relative to control. However, prodrugs were observed to be significantly cytotoxic at 250 µM. Based on these observations, all uptake and transport experiments were carried out at concentrations ≤25 µM to prevent cytotoxic effects of test compounds.

### 3.4. Cellular Uptake Studies

#### 3.4.1. Uptake of [3H]-LPV in MDCK-MDR1 and MDCK-MRP2 Cells

**Figure 2:**
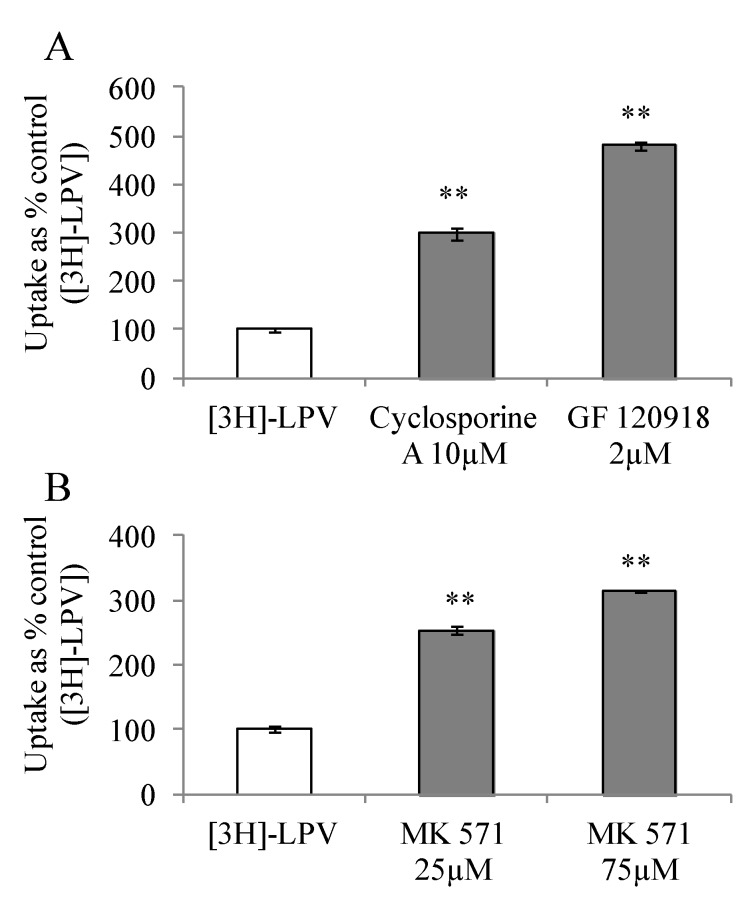
(**A**) Cellular uptake of [3H]-LPV in (A) MDCK-MDR1 cells in absence and presence of cyclosporine A (10 μM) and GF 120918 (2 μM) and (**B**) MDCK-MRP2 cells in absence and presence of MK 571 (25 and 75 μM) in DPBS (pH 7.4) at 37 °C. Each data point is expressed as mean ± standard deviation (n=4). Uptake is expressed as percentage of control ([3H]-LPV). Asterisk (**) represents significant difference from the control (*p* < 0.01).

To study affinity towards P-gp and MRP2, we carried out [3H]-LPV uptake in presence of efflux inhibitors in MDCK-MDR1 and MDCK-MRP2 cells, respectively. These cell lines represent an excellent *in vitro* cell culture model alternative to Caco-2 for high throughput drug screening [24,25]. Prior to initiation of uptake studies, cells were treated with efflux inhibitors for a period of 15 min. Uptake study was then initiated according to a procedure described in section 2.2.5. [3H]-LPV uptake increased significantly by 3 and 4.8- fold in presence of cyclosporine A and GF 120918, respectively (Figure 2A). Similarly, a 2.5 and 3- fold enhancement in the uptake process was observed in presence of 25 and 75 µM MK 571 (Figure 2B). These results clearly indicated that LPV possess excellent affinity towards P-gp and MRP2. Moreover, elevated uptake result suggested that P-gp and MRP2 are functionally active in MDCK-MDR1 and MDCK-MRP2 cells, respectively.

#### 3.4.2. Concentration Dependent Uptake Studies

**Figure 3:**
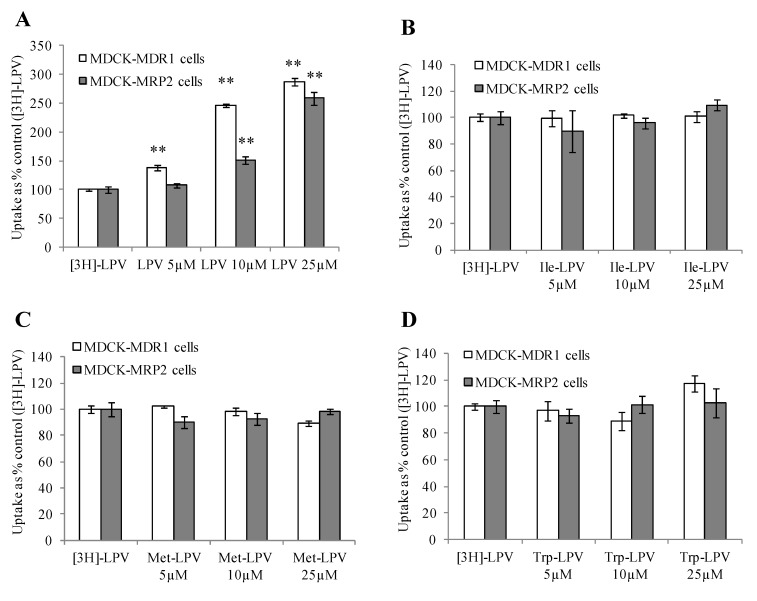
Cellular uptake of [3H]-LPV in presence of increasing concentration of LPV (A), Ile-LPV (B), Met-LPV (C) and Trp-LPV (D) in MDCK-MDR1 (empty bars) and MDCK-MRP2 (filled bars) cells in DPBS (pH 7.4) at 37 °C. Each data point is expressed as mean ± standard deviation (*n* = 4). Uptake is expressed as percentage of control ([3H]-LPV). Asterisk (**) represents significant difference from the control (*p* < 0.01).

We carried out [3H]-LPV uptake in presence of increasing concentrations of prodrugs to determine the extent of prodrug interaction with efflux proteins. As demonstrated in Figure 3A, [3H]-LPV uptake elevated significantly in presence of increasing concentrations of unlabelled LPV in MDCK-MDR1 cells. A similar trend in the enhancement of [3H]-LPV uptake in presence of increasing concentrations of unlabelled LPV was observed in MDCK-MRP2 cells (Figure 3A). These results confirmed that LPV displays significant affinity towards both P-gp and MRP2. Interestingly, [3H]-LPV uptake remained unaltered in presence of increasing concentration of prodrugs in both MDCK-MDR1 and MDCK-MRP2 cells (Figure 3B–D). These results indicated that prodrugs might have significantly lower affinity towards P-gp and MRP2 relative to LPV.

#### 3.4.3. Cellular Uptake of Unlabelled LPV and Prodrugs

To confirm the observation obtained in concentration dependent uptake studies, we carried out cellular uptake of unlabelled LPV and prodrugs in MDCK-MDR1 and MDCK-MRP2 cells. Briefly, cell monolayers were incubated with unlabelled LPV or prodrugs at 5 µM concentration for 30 min. Following incubation, LPV or prodrug solution was aspirated and the cell monolayer was washed with ice cold stop solution three times (each wash of 5 min) to terminate the uptake process. About 500 µL of DMEM was added to each well and plates were kept in −80 °C overnight for cell lysis. On the following day, LPV or prodrugs were extracted by liquid-liquid extraction technique as described in Section 3.2 and analyzed with LCMS/MS technique.

**Figure 4:**
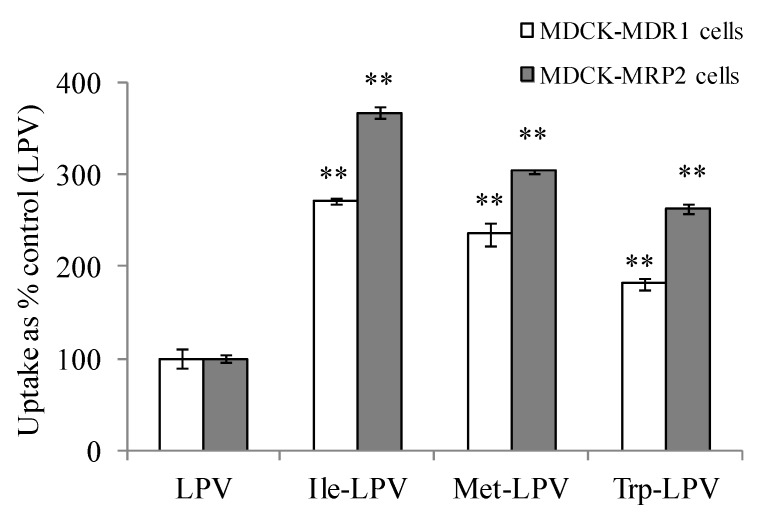
Cellular uptake of unlabelled LPV and prodrugs in MDCK-MDR1 (empty bars) and MDCK-MRP2 (filled bars) cells in DPBS (pH 7.4) at 37 °C. Each data point is expressed as mean ± standard deviation (n = 3). Uptake is expressed as percentage of control (LPV). Asterisk (**) represents significant difference from the control (*p* < 0.01).

Cellular uptake of prodrugs was observed to be significantly higher relative to LPV in both MDCK-MDR1 and MDCK-MRP2 cells (Figure 4). Ile-LPV, Met-LPV and Trp-LPV generated about 2.7, 2.4 and 1.8- fold higher uptake in MDCK-MDR1 cells relative to LPV. Similarly, uptake of Ile-LPV, Met-LPV and Trp-LPV was observed to be 3.7, 3.0 and 2.6- fold higher relative to LPV. Such uptake enhancement in MDCK-MDR1 and MDCK-MRP2 cells confirmed that prodrugs display lower affinity towards efflux proteins relative to LPV. Moreover, no enhancement in cellular uptake of prodrugs was observed in the presence of P-gp and MRP2 inhibitors (data not shown). These results confirmed that prodrug interaction with P-gp and MRP2 is significantly lower relative to LPV.

### 3.5. Transepithelial Transport Studies

To examine the effect of efflux proteins in modulating permeability of LPV and prodrugs, we carried out transepithelial transport across MDCK-MDR1 and MDCK-MRP2 cells from apical to basolateral (A-B, absorptive) and basolateral to apical (B-A, secretive) direction. In MDCK-MDR1 and MDCK-MRP2 cells, P-gp and MRP2 are expressed on the apical membrane. A classical substrate of these efflux proteins would therefore generate higher transport rate from secretive relative to absorptive direction. Hence, the role of efflux proteins in limiting LPV transport would be apparent provided there is a considerable difference in A-B and B-A permeability rates.

#### 3.5.1. LPV Transport across MDCK-MDR1 Cells

**Figure 5:**
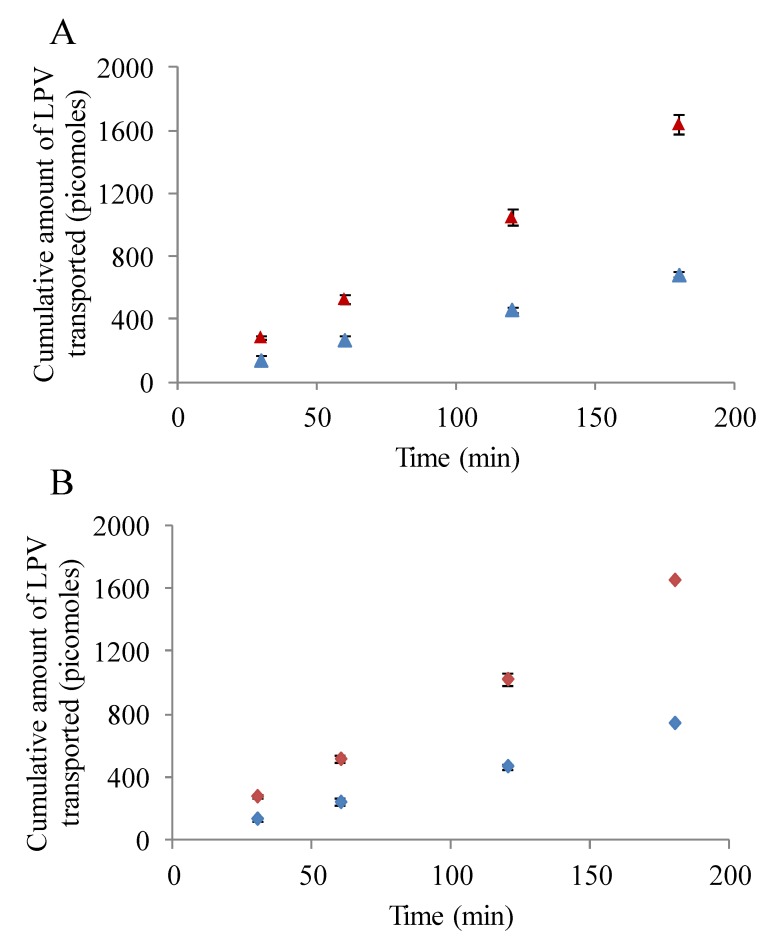
(**A**) Transepithelial bidirectional transport of LPV (25 µM) across MDCK-MDR1 cells in DPBS (pH 7.4) at 37 °C for 180 min. (**Blue** ▲) represent apical to basolateral transport (A–B) and (**Red** ▲) represents basolateral to apical transport (B-A). (**B**) Transepithelial bidirectional transport of LPV (25 µM) across MDCK-MRP2 cells in DPBS (pH 7.4) at 37 °C for 180 min. (**Blue** ♦) represent apical to basolateral transport (A–B) and (**Red** ♦) represents basolateral to apical transport (B-A). Each data point is expressed as mean ± standard deviation (n = 4).

Transepithelial LPV transport study in presence and absence of inhibitors/substrates was conducted to determine the role of P-gp in modulating the transport process. As observed in Figure 5A, transport of LPV across MDCK-MDR1 cells was significantly lower from absorptive direction relative to secretive direction. Apparent permeability of LPV form A-B and B-A directions were 2.37 ± 0.13 × 10^−^^6^ and 5.8 ± 0.5 × 10^−^^6^ cm/s, respectively. Efflux ratio displayed by LPV from B-A to A-B direction was approximately 2.5. Importantly, LPV transport increased drastically in A-B direction in presence of cyclosporine A and GF 120918 (data not shown). A-B permeability values generated by LPV in presence of cyclosporine A and GF 120918 were 8.5 ± 0.4 × 10^−^^6^ and 9.0 ± 0.3 × 10^−^^6^ cm/s, respectively. These results clearly suggest that the transport of LPV is significantly limited by P-gp.

#### 3.5.2. LPV Transport across MDCK-MRP2 Cells

Transepithelial transport of LPV across MDCK-MRP2 cells in presence and absence of MRP2 inhibitors was also carried out. Similar to previous results, LPV transport was observed to be significantly lower in A-B direction relative to B-A (Figure 5B). A-B and B-A permeability rates of LPV across MDCK-MRP2 cells were 2.7 ± 0.08 × 10^−^^6^ and 6.0 ± 0.1 × 10^−^^6^ cm/s, respectively. Efflux ratio observed was approximately 2.2. Moreover, a marked augmentation in LPV transport was observed in presence MK 571 across MDCK-MRP2 cells (data not shown). A-B LPV permeability value exhibited in presence of MK 571 was 8.7 ± 0.5 × 10^−^^6^ cm/s. Based on these observations, it is apparent that LPV transport is significantly restricted by MRP2.

#### 3.5.3. Prodrug Transport across MDCK-MDR1 and MDCK-MRP2 Cells

To investigate the potential of amino acid prodrug modification in overcoming P-gp and MRP2 mediated cellular efflux, we conducted prodrug transport across MDCK-MDR1 and MDCK-MRP2 cells. Circumvention of P-gp and MRP2 efflux mechanism would be evident provided the absorptive transport rate of prodrugs is significantly higher relative to LPV. Previously, dipeptide prodrugs of LPV have been developed and examined for their efficacy in circumventing P-gp and MRP2 mediated cellular efflux [13]. These dipeptide prodrugs displayed significantly higher A-B transepithelial transport across MDCK-MDR1 and MDCK-MRP2 cells relative to LPV. Moreover, these compounds were observed to be recognized by peptide transporters which are expressed abundantly in MDCK cells.

**Figure 6:**
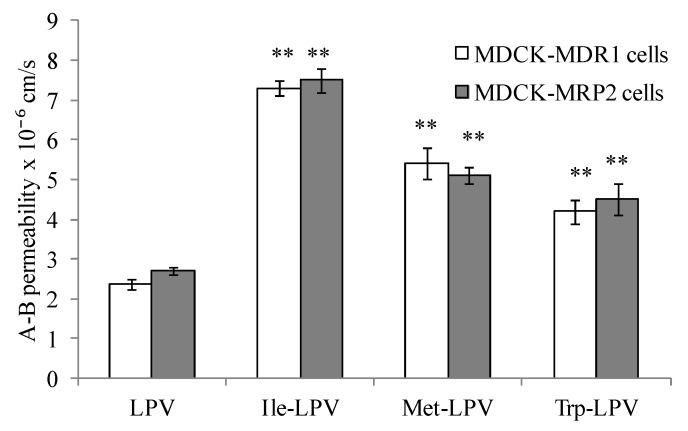
A–B permeability of LPV and prodrugs across MDCK-MDR1 and MDCK-MRP2 cells. Each data point is expressed as mean ± standard deviation (n = 4). Asterisk (**) represents significant difference from the control (LPV) (*p* < 0.01).

In this study, we examined if amino acid modified prodrugs of LPV could also circumvent P-gp and MRP-2 mediated cellular efflux. A-B permeability values obtained for LPV and prodrugs across MDCK-MDR1 and MDCK-MRP2 cells are summarized in Figure 6. Apparent permeability generated by Ile-LPV, Met-LPV and Trp-LPV across MDCK-MDR1 cells in A-B direction were 7.3 ± 0.2 × 10^−^^6^, 5.3 ± 0.4 × 10^−^^6^ and 4.2 ± 0.3 × 10^−^^6^ cm/s, respectively. A-B permeability of Ile-LPV, Met-LPV and Trp-LPV observed was about 3, 2.3 and 1.8-fold higher relative to LPV. These results indicate that prodrugs may not be recognized by P-gp relative to LPV.

Similarly, prodrugs exhibited higher A-B permeability across MDCK-MRP2 cells relative to LPV. Apparent A-B permeability generated by Ile-LPV, Met-LPV and Trp-LPV across MDCK-MRP2 cells were 7.5 ± 0.3 × 10^−^^6^, 5.1 ± 0.2 × 10^−^^6^ and 4.5 ± 0.4 × 10^−^^6^ cm/s, respectively. A-B permeability of Ile-LPV, Met-LPV and Trp-LPV was about 2.8, 1.9 and 1.7-fold higher relative to LPV. Such higher permeability values clearly suggest that prodrugs might have lower affinity towards P-gp and MRP2 relative to LPV.

To confirm this observation, we carried out A-B transport of prodrugs in presence of P-gp and MRP2 inhibitors. A–B permeability values generated by Ile-LPV, Met-LPV and Trp-LPV in presence of GF 120918 across MDCK-MDR1 cells were 6.9 ± 0.4 × 10^−^^6^, 5.8 ± 0.2 × 10^−^^6^ and 4.4 ± 0.5 × 10^−^^6^ cm/s, respectively. A-B permeability values of Ile-LPV, Met-LPV and Trp-LPV observed in presence of cyclosporine A across MDCK-MDR1 cells were 7.7 ± 0.2 × 10^−^^6^, 5.6 ± 0.3 × 10^−^^6^ and 4.1 ± 0.2 × 10^−^^6^ cm/s, respectively. Permeability values of prodrugs across MDCK-MDR1 cells were almost similar in presence and absence of GF 120918 and cyclosporine A. A-B permeability values displayed by Ile-LPV, Met-LPV and Trp-LPV across MDCK-MRP2 cells were 7.9 ± 0.7 × 10^−^^6^, 5.5 ± 0.4 × 10^−^^6^ and 4.9 ± 0.2 × 10^−^^6^ cm/s, respectively. No significant improvement in the permeability of prodrugs was observed across MDCK-MRP2 cells in presence of MRP2 inhibitor. These results confirmed that prodrugs show lower affinity towards efflux transporters such as P-gp and MRP2 relative to LPV. Based on the results obtained in the present study, we anticipate that prodrugs might produce higher oral absorption due to lower affinity towards efflux proteins and improved solubility.

### 3.6. Liver Microsomal Stability Studies

LPV is primarily metabolized by CYP3A4 enzyme. Hence, we carried out metabolic stability studies in rat liver microsomes to determine the affinity of prodrugs toward CYP3A4 relative to LPV. Previously, dipeptide prodrugs of LPV have been observed to exhibit higher metabolic stability in rat liver microsomes relative to LPV [13]. Moreover, stereoisomeric dipeptide prodrugs of saquinavir also displayed higher metabolic stability relative to saquinavir itself [26]. These results demonstrate the efficacy of dipeptide prodrugs to evade CYP3A4-mediated metabolism of LPV and saquinavir. Furthermore, sodium dependent vitamin C transporter (SVCT) targeted ascorbic acid prodrugs of saquinavir demonstrated reduced affinity towards CYP3A4 enzymes relative to parent drug, saquinavir [19]. These results clearly demonstrate the efficacy of prodrug approach in reducing CYP3A4-mediated metabolism of PIs such as LPV and saquinavir.

**Figure 7:**
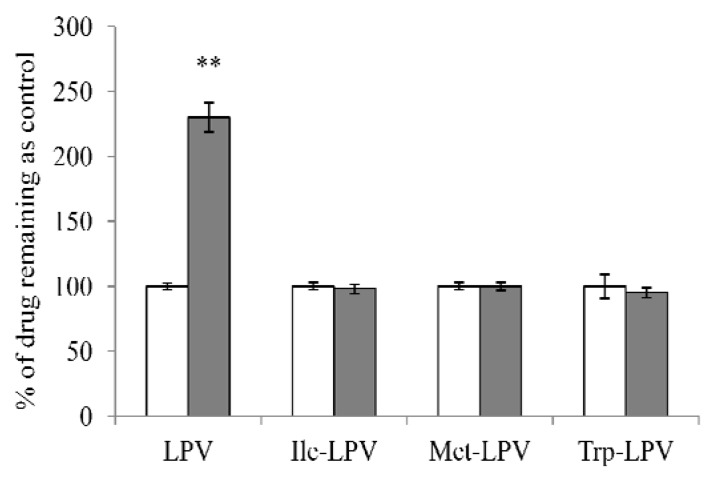
Amount of LPV or prodrug remaining after 15 min incubation in rat liver microsomes (0.3 mg/ml) in the presence (filled bars) and absence (empty bars) of ketoconazole (100 µM). Data points are expressed as mean ± SD, *n* = 4. Asterisks (**) represent significant difference from the control (in the absence of ketoconazole, *p* < 0.05).

In the present study we investigated the efficacy of amino acid prodrugs to circumvent CYP3A4-mediated metabolism of LPV. Rat liver microsomes were employed at the concentration of 0.3 mg/mL. We first determined the stability of LPV and prodrugs in liver microsomal solution in presence and absence of ketoconazole (100 µM), a potential inhibitor of CYP3A4 enzyme. Liver microsomal solution was incubated with ketoconazole for 15 min prior to the initiation of stability study. Results obtained from this study are depicted in Figure 7. In the presence of ketoconazole, amount of LPV remaining in the microsomal solution was approximately 2.3 times higher relative to control (LPV in microsomal solution without ketoconazole). This result demonstrated that LPV was extensively metabolized by CYP3A4. Interestingly, amount of each prodrug observed in microsomal solution in the presence and absence of ketoconazole was almost similar. No significant difference in prodrug concentrations was observed in the presence and absence of ketoconazole. These results indicated that prodrugs might have lower affinity towards CYP3A4 relative to LPV.

#### 3.6.1. Time Dependent Degradation Studies

We performed time dependent stability study to determine degradation rate constant in liver microsomal solution. Degradation rate constant of LPV observed was 6.0 ± 0.5 × 10^−^^3^ min^−^^1^. Interestingly, degradation rate constants displayed by prodrugs were significantly lower relative to LPV. Degradation rate constants generated by Ile-LPV, Met-LPV and Trp-LPV were about 3.8 ± 0.5 × 10^−^^3^, 3.6 ± 0.4 × 10^−^^3^ and 3.5 ± 0.2 × 10^−^^3^ min^−^^1^, respectively. These results clearly suggested that amino acid prodrugs had slightly reduced affinity for metabolic enzymes compared to LPV. Half lives of Ile-LPV, Met-LPV and Trp-LPV were observed to be approximately 1.6 to 1.8-fold higher compared to LPV. Based on this result, we anticipate that prodrugs will display higher stability in liver microsomes which in turn will result in higher systemic levels relative to LPV.

#### 3.6.2. Concentration Dependent Degradation Studies

To confirm reduced affinity of prodrugs towards metabolizing enzymes, we carried out concentration dependent degradation study by incubating various concentrations (0.5–40 µM) of LPV or prodrugs in liver microsomes (0.3 mg/mL) for 5 min. The data obtained was fitted in Michaelis-Menten kinetic model to generate Km (affinity constant) and Vmax values. Prodrugs displayed lower affinity towards metabolizing enzymes relative to LPV. Km values observed for LPV was approximately 6.0 ± 0.9 µM. Km values generated by Ile-LPV, Met-LPV and Trp-LPV were about 14.3 ± 1.1, 13 ± 2 and 13.2 ± 1.9 µM, respectively. These results confirmed previous observation that prodrugs have lower affinity towards metabolizing enzymes relative to LPV. We further determined the intrinsic clearance (Vmax/Km) of LPV and prodrugs from the data obtained in concentration dependent degradation studies. LPV displayed higher intrinsic clearance with value of 1.4 mL/min/mg protein. Ile-LPV, Met-LPV and Trp-LPV exhibited lower intrinsic clearance with values of 0.96, 0.5 and 0.77 mL/min/mg protein. Results obtained from these studies clearly confirmed that prodrugs have better metabolic stability and significantly lower affinity towards CYP enzymes compared to LPV. Thus, these amino acid prodrugs might bypass first pass effect to a significant extent relative to LPV. We also anticipate that amino acid prodrugs might generate higher systemic levels of LPV upon oral administration due to reduced metabolic degradation by CYP3A4 enzymes.

## 4. Conclusions

Amino acid prodrugs of LPV were synthesized and evaluated for their efficacy in circumventing efflux proteins and metabolizing enzymes *in vitro*. Prodrugs generated better aqueous solubility and permeability across MDCK-MDR1 and MDCK-MRP2 cells relative to LPV. Moreover, prodrugs displayed higher metabolic stability and lower affinity towards metabolizing enzymes relative to LPV. This study indicates that amino acid prodrug approach might be a viable strategy in improving the pharmacokinetic properties of LPV. Evasion of efflux process might be crucial in improving LPV transport across P-gp and MRP2 overexpressing membranes and overcoming development of resistance from chronic LPV administration. In further studies, interaction with various influx transporters such as peptide transporters, plasma protein binding and oral and brain pharmacokinetics of LPV prodrugs will be reported.

## Supplementary Files

Supplementary File 1 Supplementary Materials (DOCX, 232 KB)
