# Supplementary material for: Amino Acid Prodrugs: An Approach to Improve the Absorption of HIV-1 Protease Inhibitor, Lopinavir
Source: Pharmaceuticals (Basel). 2014 Apr 10;7(4):433–52. doi: 10.3390/ph7040433 (PMC4014701; doi:10.3390/ph7040433)
Supplement: Supplementary File 1 — Supplementary Materials (DOCX, 232 KB) [file pharmaceuticals-07-00433-s001.docx]

**Supplementary Materials**

NMR Analysis

Lopinavir

Isoleucine-LPV (Ile-LPV)

Methionine-LPV (Met-LPV)

Tryptophan-LPV (Trp-LPV)
